# Fatty Acid Modification of the Anticancer Peptide LVTX-9 to Enhance Its Cytotoxicity against Malignant Melanoma Cells

**DOI:** 10.3390/toxins13120867

**Published:** 2021-12-04

**Authors:** Fengjiao Li, Saizhi Wu, Ninglin Chen, Jingyu Zhu, Xinxin Zhao, Peng Zhang, Youlin Zeng, Zhonghua Liu

**Affiliations:** 1 The National and Local Joint Engineering Laboratory of Animal Peptide Drug Development, College of Life Sciences, Hunan Normal University, Changsha 410081, China; fengjiaoli@hunnu.edu.cn (F.L.); saizhiwu@hotmail.com (S.W.); NiinglinChen@hunnu.edu.cn (N.C.); JingyuZhu@hunnu.edu.cn (J.Z.); xinxinzhao123@hotmail.com (X.Z.); 2 Key Laboratory of Chemical Biology and Traditional Chinese Medicine Research (Hunan Normal University), Ministry of Education, College of Chemistry & Chemical Engineering, Changsha 410081, China

**Keywords:** spider venom, anticancer peptides, LVTX-9, fatty acids modification, cytotoxicity, 3D B16-F10 spheroids

## Abstract

Spider venom is a valuable resource for the development of novel anticancer drugs. In this study, we focused on novel linear amphipathic α-helical anticancer peptide LVTX-9, which was derived from the cDNA library of the venom gland of the spider *Lycosa vittata*. The cytotoxicity of LVTX-9 against murine melanoma cells in the range of 1.56–200 μM was tested and found to be significantly lower than those of most anticancer peptides reported. Its IC_50_ was determined to be 59.2 ± 19.8 μM in a serum or 76.3 ± 12.7 μM in serum-free medium. Fatty acid modification is a promising strategy for improving peptide performance. Therefore, to enhance the cytotoxic activity of LVTX-9, fatty acid modification of this peptide was performed, and five different carbon chain length lipopeptides named LVTX-9-C_12_-C_20_ were produced. Among them, the lipopeptide LVTX-9-C_18_ showed the highest cytotoxic activity in relation to B16-F10 cells, whether in a serum or serum-free medium. Most importantly, the cytotoxic activity of LVTX-9-C_18_ was improved by about 12.9 times in a serum medium or 19.3 times in a serum-free medium compared to that of LVTX-9. Subsequently, assays including scanning electron microscopy, trypan blue staining, lactate dehydrogenase leakage assay, and hemolytic activity could indicate that the potential direct cell membrane disruption is the main mechanism of LVTX-9-C_18_ to induce cancer cell death. Furthermore, the LVTX-9-C_18_ also showed strong cytotoxicity in relation to 3D B16-F10 spheroids, which indicates it might be a promising lead for developing anticancer drugs.

## 1. Introduction

Despite current scientific and technological progress as well as the continuous improvement of medical standards, cancer remains a major public health problem worldwide, and the mortality rate for cancer is also increasing year to year [1]. It is a challenge to exert long-term anticancer effects using radiotherapy and chemotherapy, which may also produce serious side effects and drug resistance. Therefore, it is particularly important to exploit novel, safe, and efficient anticancer drugs. Anticancer peptides (ACPs) and their derivatives have a broad anticancer spectrum and strong and rapid anticancer effects, which means they can be considered good candidate precursor molecules for cancer therapy. Natural toxins from venomous animals such as snakes, spiders, bees, scorpions, and conus, are precious resources for developing novel anticancer drugs [2,3,4,5,6,7,8]. For instance, lycosin-I, isolated from the spider *Lycosa singoriensis*, with a linear amphipathic alpha-helical conformation, can effectively suppress tumor growth in vitro and in vivo [9]. Melittin, derived from the bee venom of *Apis mellifera*, can inhibit growth factor receptor activation in HER2-enriched and triple-negative breast cancer [10]. Nevertheless, there are some disadvantages to ACPs, such as low stabilization and cytotoxicity, which limit the development of cancer drugs. To improve the performance, many ACPs have been modified to become candidates for drug development with higher activity, better stability, etc. Various modification strategies, such as amino acid substitution [11], polyethylene glycol (PEG) modification [12,13], fatty acid modification [14,15], glycosylation [16,17], etc., have been exploited to improve a variety of properties of ACPs.

It has been reported that fatty acid modification has great potential for optimizing peptide function. For example, PepFect14, a cell-penetrating peptide, was modified with fatty acids of different lengths, improving the delivery efficiency and increasing the splice-correcting activity [18]. Antimicrobial peptides conjugated with fatty acids exhibited improved anti-tumor activity and multi-drug resistance-reversing effects. Furthermore, the conjugation of aliphatic acid also enhanced peptide stability in plasma [19]. The conjugation of fatty acids at the N-terminus of antimicrobial agents also presented high stability in the presence of protease or serum [20]. Surprisingly, the N-terminal modification of fatty acids can also greatly enhance the cytotoxic activity of anticancer peptides. For instance, the cytotoxicity of R-C_16_ was found to be 3~4-fold higher than R-lycosin-I [14]. Similarly, the lipopeptide L-C_12_ exhibited stronger cytotoxicity than lycosin-I (8~9 times) [21].

**Figure 1:**
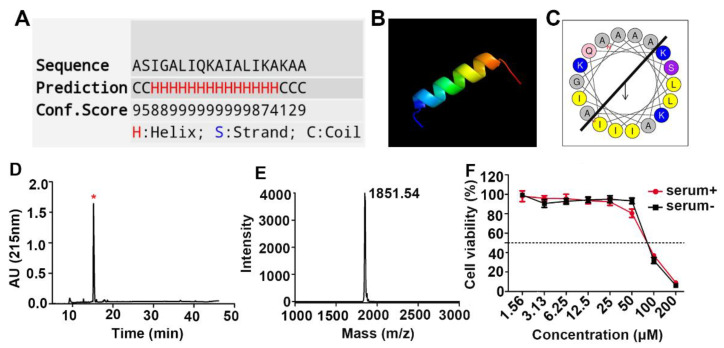
The characterization of the peptide LVTX-9: Table of prediction results of secondary structure of LVTX-9 (**A**) and diagram of the secondary structure model of LVTX-9 (**B**) using i-Tasser software. The red H indicates that the secondary structure is a helix. The blue S shows that the secondary structure is a strand. The black C indicates that the secondary structure is a coil. Conf. score presents the credibility of the prediction. Helical wheel structure of LVTX-9 was predicted by HeliQuest (https://heliquest.ipmc.cnrs.fr/cgi-bin/ComputParams.py, accessed on 25 July 2021) (**C**). Purification of LVTX-9 using RP-HPLC and the red star (*) represents the target peak (**D**). Identification of LVTX-9 using MALDL-TOF MS, and the molecular mass is 1851.54 (**E**). The cell viability of LVTX-9 on B16-F10 cells in serum-containing and non-serum-containing medium (**F**).

*Lycosa vittata*, a medium-sized and venomous spider, is chiefly found in the southwest of China [22]. The bioactivity of many components of its venom remains unreported. In a previous study, according to the cDNA library of a *Lycosa vittata* venom gland that was kept in our laboratory (unpublished data), we designed many linear and amphipathic peptides based on the relevant characteristics of anticancer peptides. For example, α-helix anticancer peptide LVTX-8 exhibited strong anticancer activity both in vitro and in vivo [23]. Similar to LVTX-8, LVTX-9 was also derived from the cDNA library of the *Lycosa vittata* venom gland and exhibited characteristics of ACPs such as its linear nature, α-helix conformation, amphipathicity, etc. [24,25] (Figure 1). However, its cytotoxicity in relation to cancer cells was significantly lower than that of ACPs that have been reported such as LVTX-8, melittin, etc. 

The purpose of this study was to exploit the fatty acid modification of LVTX-9 to enhance its cytotoxicity against cancer cells. Therefore, LVTX-9 and five lipopeptides, LVTX-9-C_12_, LVTX-9-C_14_, LVTX-9-C_16_, LVTX-9-C_18_, and LVTX-9-C_20_, were successfully synthesized. To some extent, increasing hydrophobicity can enhance the cytotoxic activity of anticancer peptides, as hydrophobicity controls the extent to which ACPs can partition into the membrane layer. Therefore, we hypothesized that adding hydrophobic fatty acids would significantly enhance the cytotoxicity of LVTX-9. To test this hypothesis, a CCK-8 assay, various physical and chemical characterizations, scanning electron microscopy (SEM), trypan blue staining, a lactate dehydrogenase (LDH) leakage assay, and the determination of hemolytic activity were carried out to investigate the cytotoxicity and the membrane disruption of the lipopeptide. Finally, we constructed B16-F10 tumor spheroids to further evaluate its toxicity at the 3D cellular level.

## 2. Results

### 2.1. Design, Synthesis, and Cytotoxicity Characterization of the Anticancer Peptide LVTX-9 

This designed peptide consisted of 19 residues, i.e., ASIGALIQKAIALIKAKAA. A prediction of its secondary structure and peptide helical wheel showed that LVTX-9 is a linear and amphipathic α-helical peptide (Figure 1A–C). Exploiting SPPS and RP-HPLC, we successfully synthesized and purified LVTX-9 (Figure 1D). In addition, MALDI-TOF MS determined the sample had a molecular mass of 1851.54 Da, which was consistent with the theoretical molecular weight of 1851.28 Da (Figure 1E). Then, B16-F10 cells were chosen to investigate the cytotoxic activity of LVTX-9 using a CCK-8 assay. As shown in Figure 1F, LVTX-9 exhibited low cytotoxic activity with or without serum in B16-F10 cells. The CCK-8 assay indicated that, as an anticancer peptide, the cytotoxicity of LVTX-9 needs improvement.

### 2.2. Fatty Acid Modification of Anticancer Peptide LVTX-9 

**Scheme 1:**
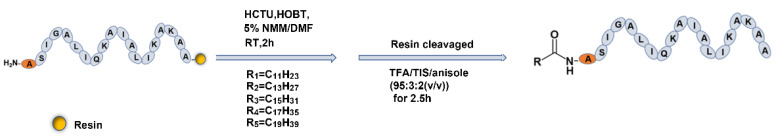
Preparation route of five lipopeptides.

**Figure 2:**
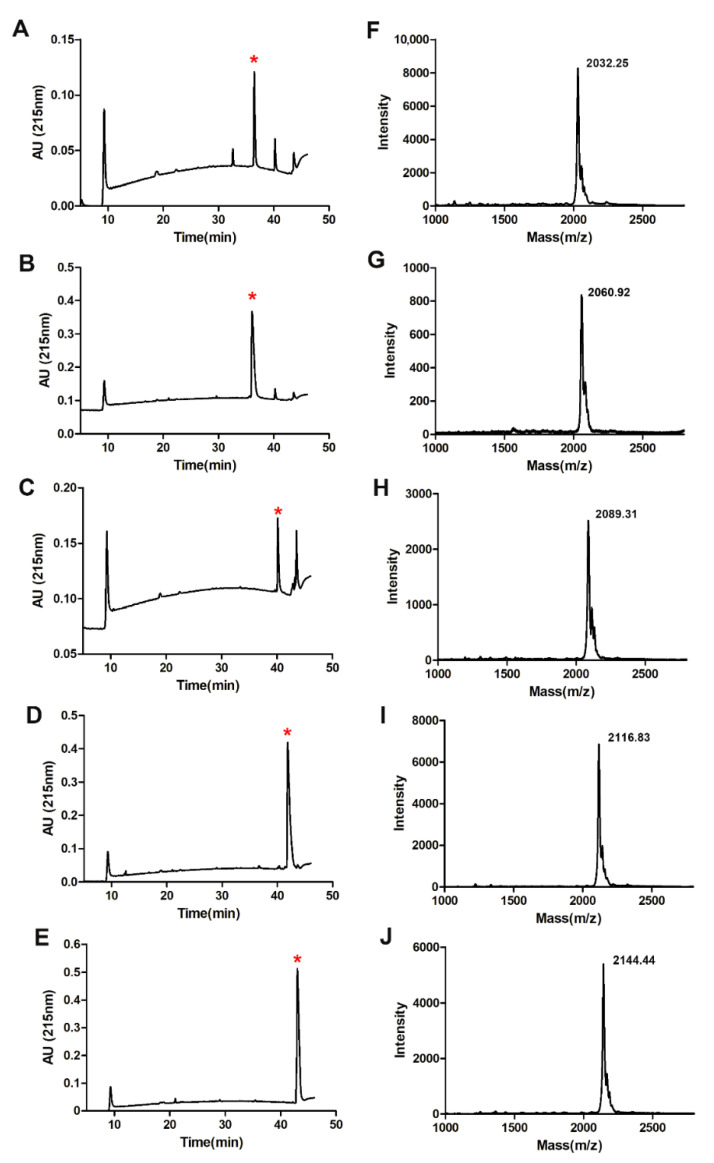
Purification and identification of five lipopeptides using RP-HPLC and MALDL-TOF MS: The purification of (**A**) LVTX-9-C_12_, (**B**) LVTX-9-C_14_, (**C**) LVTX-9-C_16_, (**D**) LVTX-9-C_18_, and (**E**) LVTX-9-C_20_ was performed using RP-HPLC (column, Vydac, C18, 300 Å, 4.6 × 250 mm). The lipopeptides were separated at a flow rate of 1.0 mL/min under a gradient of 30–100% acetonitrile/0.1% TFA. The elution of lipopeptides was monitored at 215 nm. Identification of (**F**) LVTX-9-C_12_, (**G**) LVTX-9-C_14_, (**H**) LVTX-9-C_16_, (**I**) LVTX-9-C_18_, and (**J**) LVTX-9-C_20_ by MALDI-TOF MS, respectively. The red star (*) represents the target peak.

Here, we adopted a fatty acid modification strategy to enhance the cytotoxicity of the peptide LVTX-9. As shown in Scheme 1, different lengths of fatty acid were coupled with the N-terminal of LVTX-9 in 5% NMM/DMF with HCTU and HOBT at room temperature (RT) for 2 h. The lipopeptide chains were cleaved from resin in TFA/TIS/anisole (95:3:2 (*v*/*v*)) for 2.5 h. The corresponding lipopeptides were named LVTX-9-C_12_, LVTX-9-C_14_, LVTX-9-C_16_, LVTX-9-C_18_, and LVTX-9-C_20_ based on the length of the carbon chain (Table 1). RP-HPLC was used to purify the synthesized lipopeptides (Table S1, Figure 2A–E). The retention times of LVTX-9 and the five lipopeptides indicate that the fatty acid chain length was positively correlated with the hydrophobicity of the lipopeptides. The overall order of hydrophobicity is LVTX-9-C_20_ > LVTX-9-C_18_ > LVTX-9-C_16_ > LVTX-9-C_14_ ≈ LVTX-9-C_12_ > LVTX-9 (Table 1). MALDI-TOF MS was used to determine the relative molecular masses of the lipopeptides (Figure 2F,J). These results show that the actual molecular weight was consistent with the theoretical molecular weight; the red star represents the target peak.

### 2.3. Cytotoxicity of Lipopeptides

**Figure 3:**
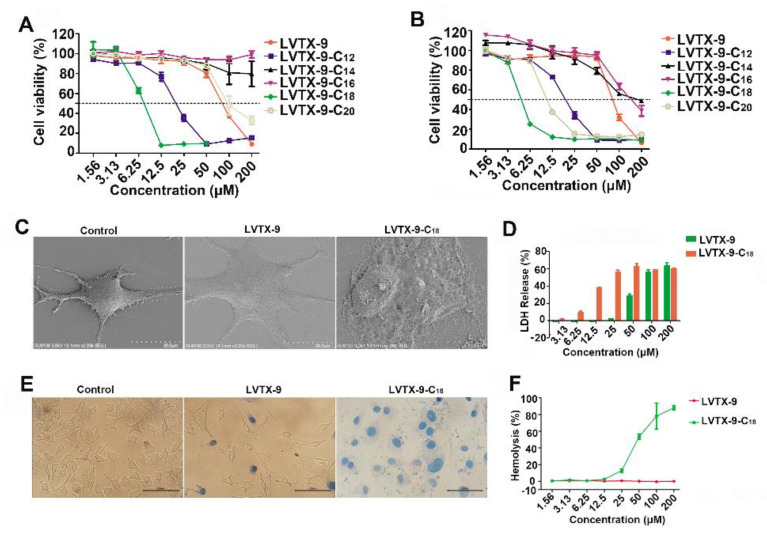
Characterization of bioactivity of the lipopeptides: Cytotoxic activities of LVTX-9 and five lipopeptides in B16-F10 cells in serum-containing (**A**) or serum-free (**B**) medium as determined by CCK-8 assay (*n* = 3). (**C**) SEM images of B16-F10 cells after treatment with 50 μM of LVTX-9 or 5 μM of LVTX-9-C_18_. (**D**) LDH leakage measurement after B16-F10 cells were treated with LVTX-9 or LVTX-9-C_18_ at different concentrations for 24 h (*n* = 3). (**E**) Trypan blue staining images. (**F**) Hemolytic activity of LVTX-9 and LVTX-9-C_18_ against erythrocytes. Drug concentrations ranged from 1.56 to 200 µΜ. The absorbance of the supernatant was detected by microplate reader at 450 nm.

B16-F10 cells were chosen to determine the cytotoxic activity of five lipopeptides using a CCK-8 assay. Interestingly, different lipopeptides exhibited greatly different cytotoxic activities in B16-F10 cells compared to LVTX-9 (Figure 3A,B). The IC_50_ values of LVTX-9, LVTX-9-C_12_, LVTX-9-C_14_, LVTX-9-C_16_, LVTX-9-C_18_, and LVTX-9-C_20_ in serum-containing medium were 59.2 ± 19.8 μM, 15.7 ± 3.5 μM, >200 μM, >200 μM, 8.5 ± 2.9 μM, and ≈100 μM, respectively, but were 76.3 ± 12.7 μM, 13.4 ± 5.3 μM, >100 μM, >100 μM, 4.4 ± 0.2 μM, and 11.16 ± 0.8 μM in serum-free medium, respectively. In addition, the cytotoxicity of the five lipopeptides in L-929 cells was very similar to that of B16- F10 cells, indicating they have no selectivity (Figure S1). LVTX-9-C_18_ showed the highest cytotoxicity in B16-F10 cells among the five lipopeptides, both in the serum-free and the serum-containing medium, and so it was chosen in follow-up experiments. Similarly, the IC_50_ values of LVTX-9 against various cancer cells (4T1, HepG2, and Hela) except for A549 (IC_50_ ≈ 51 μM) in serum-free medium, were >100 μM. The results also show that LVTX-9 exhibits very low cytotoxicity. Meanwhile, the IC_50_ values of LVTX-9-C_18_ against various cancer cells ranged from 3.6–7.6 μM, which further confirmed that octadecanoic acid conjugation greatly enhanced the cytotoxic activity of LVTX-9 (Table 2). 

In this study, various assays were performed to explore the action mechanism of LVTX-9-C_18_. In this part, 5 μM—which is the IC_50_ value of LVTX-9-C_18_ (Figure 3B)—was selected to perform SEM and trypan blue staining. In the experiments, 50 μM, which is 10 times the IC_50_ value of LVTX-9-C_18_, was selected as the treatment concentration of LVTX-9. As the control, 50 μM of LVTX-9 had no significant effect on cell viability after 24 h of treatment (Figure 3B). Furthermore, if the anticancer effect of a high concentration of LVTX-9, such as the selected amount of 50 μM, was significantly less than that of a low concentration of LVTX-9-C_18_, such as 5 μM, this also indicates that fatty acid modification increased the activity of LVTX-9. As shown in Figure 3C, the results of SEM indicate that untreated or 50 μM of LVTX-9-treated B16-F10 cells had intact membranes with smooth surfaces exhibiting a plump spindle cell morphology. However, cells treated with 5 μM of LVTX-9-C_18_ had a membrane that was totally disorganized or even absent, and membrane disruption was indicated. Membrane rupture was often accompanied by the release of LDH; therefore, membrane integrity was generally evaluated by detecting LDH release [26]. As shown in Figure 3D, LVTX-9-C_18_ contributed to intense LDH release from B16-F10 cells. For example, 12.5 μM of LVTX-9-C_18_ caused the release of ~40% LDH, but 25 μM of LVTX-9 only led to the release of ~2% LDH. Thus, these results demonstrate that LVTX-9-C_18_ could exert strong membrane-lytic activity compared to LVTX-9. Trypan blue staining indicates that the control or 50 μM of LVTX-9-treated B16-F10 cells presented as almost unstained, but cells treated with 5 μM of LVTX-9-C_18_ were stained blue (Figure 3E). Only when the cell membrane is damaged does trypan blue enter the cell and stain the cell blue. Therefore, these results further suggest that LVTX-9-C_18_ can cause the loss of cell membrane integrity. Finally, hemolytic activity was also used to assess the membrane-lytic activity of LVTX-9-C_18_. As shown in Figure 3F, 200 μM of LVTX-9 showed almost no hemolytic activity. LVTX-9-C_18_ only induced about 10% hemolysis at 25 µM, but over 25 µM, it demonstrated stronger hemolytic activity. In short, the LDH release assay, trypan blue staining, and hemolytic assessment indicate that LVTX-9-C_18_ can disrupt the cell membrane and then induce cell death.

### 2.4. Anti-Proliferative Effect of LVTX-9 and LVTX-9-C_18_

**Figure 4:**
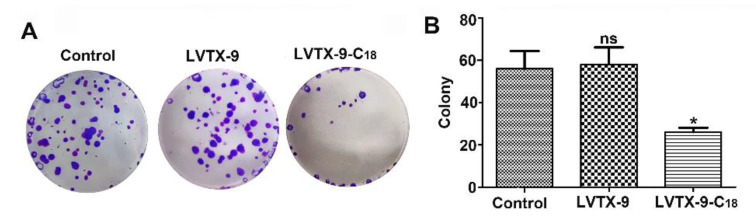
Anti-proliferative effect of LVTX-9 and LVTX-9-C_18_: (**A**) Colony formation viability of B16-F10 cells treated with 10 μM of LVTX-9 and 1 μM of LVTX-9-C_18_ was evaluated by colony formation assay. (**B**) Quantitative results are illustrated for clonogenic assay (*n* = 3). Statistical analysis was performed using GraphPad software 5.0 (One-way ANOVA test). ns, non-significant, * *p* < 0.05.

Based on our previous study [21], the concentration of cytotoxic peptide used in colony formation testing must be lower than the IC_50_ which can be calculated via a CCK-8 assay, as colony formation primarily tests the anti-proliferation of drugs rather than their toxic effects. Therefore, 1 μM of LVTX-9-C_18_, which had no significant cytotoxic effect on B16-F10 cells, was selected as the test concentration for clone formation. Similarly, 10 μM of LVTX-9, which is also 10 times the concentration of LVTX-9-C_18_, was chosen as the control. As shown in Figure 4A,B, the colony number of the 10 μM of LVTX-9-treated group (58 ± 11.4) was similar to that of the control group (51 ± 12.0). However, the colony number of the 1 μM of LVTX-9-C_18_-treated group (26 ± 2.9) was a significantly different from that of the control group (*p* = 0.0198). Compared with 10 μM of LVTX-9, 1 μM of LVTX-9-C_18_ exhibited a strong antiproliferative effect, as determined by a colony formation assay. Among the five fatty acids, octadecanoic acid modification (LVTX-9-C_18_) had the greatest enhanced cancer cell proliferation inhibition at low concentrations (e.g., 1 μM). 

### 2.5. Physicochemical Characteristics of LVTX-9 and LVTX-9-C_18_

**Figure 5:**
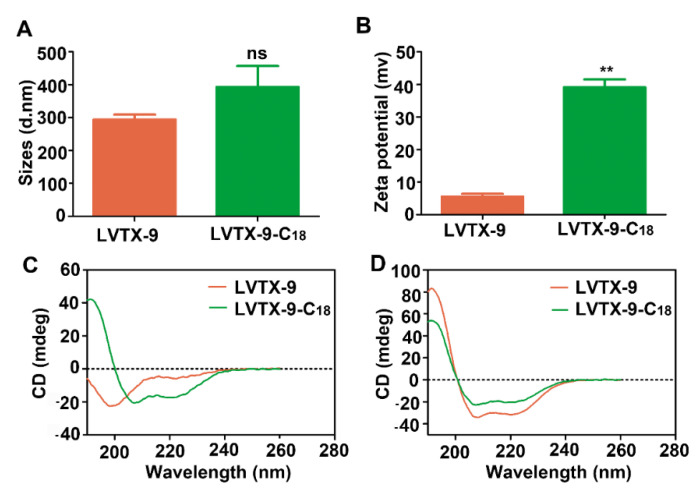
Physical characteristics of LVTX-9 and LVTX-9-C_18_: (**A**) The size and zeta potential (**B**) of LVTX-9 and LVTX-9-C_18_ analyzed using DLS. (**C**) The secondary structure of LVTX-9 and LVTX-9-C_18_ were measured in ultrapure water at 25 °C, and (**D**) 100 mM of SDS at 25 °C by CD spectrometer. Statistical analysis was performed using GraphPad software 5.0 (*t*-test). ns, non-significant, ** *p* < 0.01.

To illustrate the greatly enhanced cytotoxicity of LVTX-9-C_18_, the physical and chemical characteristics of LVTX-9 and LVTX-9-C_18_ were analyzed and compared, as these play a key role in the cytotoxicity of anticancer peptides. As shown in Figure 5A, the hydrodynamic size of LVTX-9-C_18_ (PDI = 0.64 ± 0.1369) was similar to that of LVTX-9 (PDI = 0.65 ± 0.0481). Interestingly, the zeta potential of LVTX-9-C_18_ was significantly higher than that of LVTX-9 (*p* = 0.0084, Figure 5B). In addition, the circular dichroism (CD) spectrum was determined in ultrapure water and 100 mM of SDS. As shown in Figure 5C,D, LVTX-9 presented random coil conformation, but LVTX-9-C_18_ showed α-helical conformation in ultrapure water. In SDS, they all exhibited α-helical conformation.

According to the physical and chemical characteristics, we explain the reasons for the great improvement in cytotoxicity of LVTX-9-C_18_ as follows: the addition of octadecanoic acid increased the zeta potential of LVTX-9, which may have enhanced the ability of the peptide to bind to the cancer cell membrane. Furthermore, the greatly enhanced hydrophobicity (Table 1) and helical conformation increased the partition and insertion of LVTX-9 into cancer cell membranes. 

### 2.6. LVTX-9-C_18_ Exhibits Cytotoxicity in Relation to B16-F10 Tumor Spheroids

**Figure 6:**
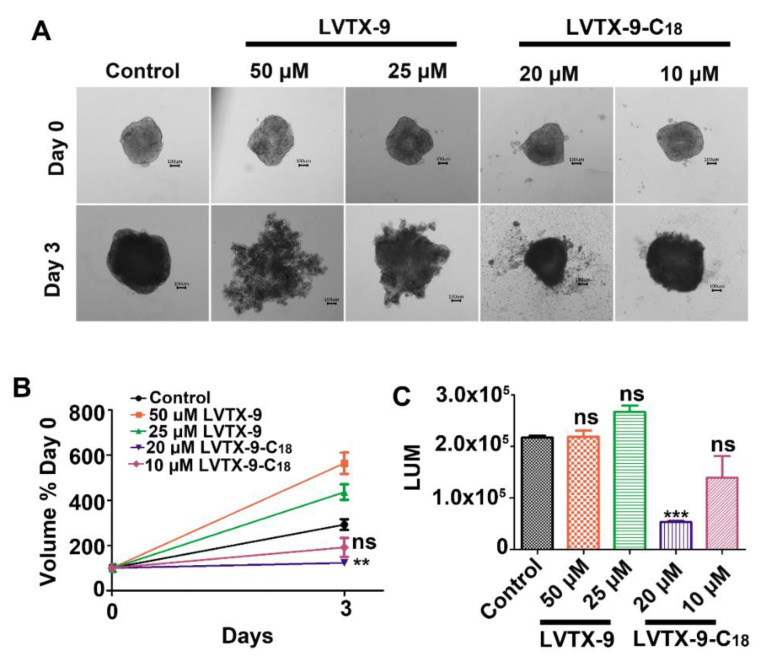
Cytotoxicity effects of LVTX-9 and LVTX-9-C_18_ on B16-F10 tumor spheroids: (**A**) Tumor spheroid images on day 0 and day 3. (**B**) Volume quantitative results of tumor spheroids. (**C**) Cell viabilities of the tumor spheroids were obtained in the presence of different concentrations of LVTX-9 and LVTX-9-C_18_ by CellTiter-Glo 3D Cell Viability Assay, respectively (*n* = 4). Statistical analysis was performed using GraphPad software 5.0 (One-way ANOVA test). ns, non-significant, ** *p* < 0.01, and *** *p* < 0.001.

The concentrations of LVTX-9-C_18_ were chosen for research on its cytotoxicity in relation to B16-F10 tumor spheroids based on two points: (1) The first is our previous study [16,21], in which we also tested the cytotoxic effect of lipopeptide L-C_12_ on tumor spheroids. This provided some reference for us in choosing the concentrations. (2) The second is the properties of 3D tumor spheres. Tumor spheroids have a three-dimensional, compact, spherical structure. Peptides need to penetrate the tumor spheroids and then exhibit cytotoxicity. This means that the concentrations we selected needed to be higher than those for cytotoxicity tests in 2D cells. As such, to better determine the concentration of LVTX-9-C_18_ acting on tumor spheres, two concentrations (20 and 10 μM) were chosen, and 50 and 25 μM of LVTX-9 were selected as controls. B16-F10 tumor spheroids were treated with LVTX-9 and LVTX-9-C_18_, and spheroid images and volumes were recorded and measured on days 0 and 3. In the control and LVTX-9 groups, the spheroids gradually grew, while the growth of the spheroids was significantly suppressed with the treatment of 20 μM of LVTX-9-C_18_ (*p* = 0.0072, Figure 6A,B). Interestingly, LVTX-9 has a dispersive effect on the 3D tumor sphere, which results in the calculated volume being larger than that of the control. Compared with the control and LVTX-9, 20 μM of LVTX-9-C_18_ notably reduced the cell viability of the spheroids as determined by a CellTiter-Glo 3D Cell Viability Assay (*p* < 0.0001, Figure 6C). Overall, LVTX-9-C_18_ exhibited significant cytotoxicity on B16-F10 tumor spheroids, which might be related to its greatly enhanced cytotoxicity; this further confirmed that LVTX-9-C_18_ could represent an excellent lead molecule for anticancer drugs.

## 3. Discussion and Conclusions

Peptide therapeutics have gained attention and come to be gradually approved in the medical field since the advent of insulin therapy, which enters clinical development at a steady pace [27,28]. Animal venoms have always represented a valuable resource of leading molecules for anti-tumor active drugs [29]. Here, we designed a novel toxin peptide, termed LVTX-9, derived from the *Lycosa vittata*’s venom gland cDNA library, based on properties of anticancer peptides, such as linear, α-helix conformation, amphipathicity, etc. Although LVTX-9 has the characteristics of anticancer peptides, it demonstrated low cytotoxic activity in cancer cells. To date, strategies such as glycosylation [17], covalent coupling modification [30], and combination therapy [31] have been applied to greatly improve the performance of peptides. Among these strategies, fatty acid modification is a widely used and effective way of optimizing of anticancer drugs. In this study, fatty acid modification was performed by different lengths of carbon chain covalently linked to the *n*-terminal of LVTX-9. 

First, compared to LVTX-9, five lipopeptides exhibited different cytotoxicity profiles. The modified carbon chain length was positively correlated with hydrophobicity. Generally speaking, with the increase of the length of carbon chain, the biological activity of the fatty acid-modified peptide first increased and then decreased, with an optimum hydrophobicity window in which a high level of biological activity could be obtained. Among the five synthesized lipopeptides, LVTX-9-C_18_ exhibited the strongest cytotoxicity against cancer cells among the five lipopeptides, which indicates that its hydrophobicity was probably at an optimal level. Even so, LVTX-9-C_18_ demonstrated strong cytotoxicity to non-cancer cells, indicating that it has no selectivity.

LVTX-9-C_18_ was chosen in follow-up physicochemical characterization experiments. The overall net charge and hydrodynamic size are important factors for ACPs to disrupt cell membranes, as they can affect the binding ability of cationic ACPs to the negatively charged phospholipid component of cancer cell membranes through electrostatic interactions [32,33]. In addition, hydrophobicity controls the extent to which ACPs can partition into the membrane layer. [34]. Furthermore, helical conformation is often required for ACPs to penetrate or be inserted into cell membranes [35]. Therefore, as an ACP, the enhanced zeta potential and hydrophobicity of LVTX-9-C_18_ may have contributed to its highly increased cytotoxicity, due to an enhanced binding ability and the great extent to which it can partition into the membrane layer.

Then, in our study, we found that LVTX-9-C_18_-induced cell death may occur in two ways, as follows: On the one hand, when the concentration is above 3 μM for LVTX-8-C_18_ (Figure 3B), potential direct cell membrane disruption may be the main action mechanism for ACPs inducing cell death (Figure 4). The cytotoxic activity of LVTX-8-C_18_ is mainly expressed as the potential direct cell membrane disruption, and the membrane disruption effect is rapid [36]. On the other hand, LVTX-9-C_18_ can also suppress colony formation at a lower concentration (1 μM), indicating that LVTX-9-C_18_ can also inhibit cell proliferation. Our previous study demonstrated that lycosin-I, a linear amphipathic α-helical anticancer peptide, can upregulate p27 to inhibit cell proliferation [9]. As LVTX-9 exhibits certain properties that are similar to lycosin-I, such as being linear, amphipathic, and α-helical, we speculated that upregulation of p27 expression may be one of the ways in which LVTX-8-C_18_ inhibits cell proliferation.

Finally, 3D tumor spheroids are heterogeneous cellular aggregates with hypoxic regions and necrotic centers, which can be used as 3D tumor models for standardized and rapid cancer function research [37,38]. A cytotoxicity assay of 3D tumor spheroids demonstrated that LVTX-9-C_18_ exhibited potent cytotoxicity against tumor spheroids in vitro. Extensive evidence shows that in vitro 3D tumor models can more accurately reproduce gene expression profiles, signaling pathway activity, and drug sensitivity than simple 2D cell monolayers [39]. Therefore, we speculated that LVTX-9-C_18_ showing efficacy in 3D tumor spheroids may indicate that it is also effective in animal models. Taken together, our work demonstrates that fatty acid modification is an effective strategy to enhance the cytotoxicity of anticancer peptide LVTX-9, and that LVTX-9-C_18_ might be a promising lead for developing anticancer drugs.

## 4. Materials and Methods

### 4.1. Reagents

All Fmoc amino acids, rink amide-AM resin, HCTU, and HOBT were purchased from GL Biochem (Shanghai, China). The cell counting Kit-8 (CCK-8) and LDH cytotoxicity assay kit were order from Beyotime (Shanghai, China). Crystal violet and trypan blue were obtained from Sigma-Aldrich.

### 4.2. Cell Cultures

Mouse skin melanoma cells (B16-F10) were cultured in RPMI-1640 (Gibco), and mouse fibroblast cells (L-929), human lung carcinoma cells (A549), mouse breast cells (4T1), human hepatocarcinoma cells (HepG2), and human cervical carcinoma cells (Hela) were cultured in Dulbecco’s Modified Eagle Medium (DMEM, Gibco). All culture medium contained 10% fetal bovine serum (serum, Gibco), 100 U/mL of penicillin, and 100 mg/mL of streptomycin at 37 °C with 5% CO_2_.

### 4.3. Synthesis, Purification, and Identification of LVTX-9 and Five Lipopeptides

Peptides were synthesized using the Fmoc SPPS methods [40]. Briefly, the peptide LVTX-9 (AC-ASIGALIQKAIALIKAKAA-amide) was prepared on Rink Amide AM resin at a 0.2 mmol scale. A 4-fold excess of the Fmoc-protected amino acids was coupled with the Rink Amide AM resin using HCTU and HOBT as the activating agents in 5% NMM/DMF. After the synthesis of LVTX-9, the N-terminal Fmoc group was removed using 20% piperidine. As in the previous steps, a series of fatty acids (C_12_-C_20_) was activated in HCTU and HOBT using 5% NMM/DMF, and coupled to peptidyl resin for 2 h. Peptides were cleaved using trifluoroacetic acid (TFA)/thioanisole (TIS)/anisole (95:3:2 (*v*/*v*)) for 2.5 h, and the collected lysate was precipitated in cold diethyl ether three times. The precipitate was air-dried naturally. Next, the obtained crude products were purified using RP-HPLC (C18, 4.6 mm × 250 mm) on the basis of a 0.1% TFA/acetonitrile gradient from 30% to 100% in 46 min. Then, we collected the eluted peptides and determined the molecular mass using MALDI-TOF MS (AB, SCIEX, Waltham, MA, USA).

### 4.4. Cell Viability Assays

Cell viability was quantified using a CCK-8 assay [9]. At first, cells were seeded into a 96-well plate at approximately 2 × 10^3^ cells/well. The following day, we created a 2-fold dilution series of each peptide using fresh medium (with or without 10% serum) for the treated cells, and cell viability was detected at 450 nm via a CCK-8 assay using an Absorbance Microplate Reader (BioTek Instruments, Winooski, VT, USA) following 24 h of drug incubation. The activity curves and IC_50_ values of the peptides were visualized using GraphPad prism 5.0.

### 4.5. Scanning Electron Microscopy

The morphologic changes of the cell membrane of B16-F10 cells treated with peptides were observed by SEM [14]. The cells were seeded on coverslips and placed into a six-well plate, then treated with LVTX-9 (50 μM) and LVTX-9-C_18_ (5 μM) for 24 h. After incubation, the B16-F10 cells were washed with sterile PBS gently three times and fixed with 2 mL of 2.5% glutaraldehyde solution in each well for 4 h at room temperature. Then, the fixed cells were dehydrated with a series of ethanol concentrations. We dried the samples with a Critical Point Dryer and coated them with gold plating before observation. A scanning electron microscope (Hitachi Su8010, Tokyo, Japan) was used to examine the prepared samples.

### 4.6. Physical Characterization of Peptides

Dynamic light scattering (DLS) of Zetasizer Nano ZSP (Malvern Instruments, Malvern, UK) apparatus was used to determine the hydrodynamic size and zeta potential of LVTX-9 and LVTX-9-C_18_. The peptides concentrations were diluted to 100 µM with ultrapure water with a final volume of 1 mL. The procedure ran at 25 °C, with each experiment repeated three times. The secondary structures of LVTX-9 and LVTX-9-C_18_ were determined to be in line with the previous protocol [11]. The peptide concentrations were then diluted to 100 µM with ultrapure water and 100 mM of SDS was added in a final volume of 250 μL. The procedure was carried out at 25 °C. CD spectra were recorded between 180 nm and 280 nm on a Jasco J-815 CD spectrometer.

### 4.7. LDH Leakage Assays

At first, the B16-F10 cells were cultured in 96-well plates for 24 h at approximately 2 × 10^3^ cells/well. Subsequently, the cells were treated for 24 h with LVTX-9 or LVTX-9-C_18_ with a series of concentration gradients. LDH leakage detection was detected using an LDH Cytotoxicity Assay Kit according to the manufacturer’s instructions [26] (Beyotime, Shanghai, China. C0017). The absorption was measured using an Absorbance Microplate Reader (BioTek Instruments) at 490 nm. The LDH release (%) of peptides was calculated by GraphPad prism 5.0.

### 4.8. Colony Formation Assay

To detect the proliferation of B16-F10 cells treated with LVTX-9 and LVTX-9-C_18_ at low concentrations, digested cells were counted using a Cellometer K2 and seeded into a 12-well plate with approximately 100 cells per well. After 36 h, cells were treated with LVTX-9 (10 μM) and LVTX-9-C_18_ (1 μM), then incubated for 7 days at 37 °C in a humidified 5% CO_2_ atmosphere. Visible colonies were stained with 0.5% crystal violet and photographically recorded. The colony formation rates were calculated, and curves were manufactured by GraphPad prism 5.0.

### 4.9. Trypan Blue Staining

B16-F10 cells were seeded into a 48-well plate (2 × 10^3^ cells/well) for 24 h and treated with LVTX-9 (50 μM) and LVTX-9-C_18_ (5 μM) for 24 h. The cells were washed with PBS gently and stained with 0.04% Trypan blue for 4 min. Dead cells were stained blue.

### 4.10. Determination of Hemolytic Activity

The hemolytic activity levels [18,41] of LVTX-9 and LVTX-9-C_18_ were confirmed as follows. Red blood cells (RBC) were washed with sterile PBS and centrifuged for 5 min at 1000 rpm. The supernatant was removed, and cells were resuspended in PBS at a final concentration of 1% (5 × 10^6^/mL). The diluted RBC suspension (50 µL) was mixed with PBS (negative control), 0.1% Triton-X 100 (positive control), and LVTX-9 or LVTX-9-C_18_ with various concentrations at a 1:1 ratio. The mixtures were incubated for 30 min at 37 °C, then centrifuged for 5 min at 12,000 rpm. The supernatant (90 µL) was added to a new 96-well plate to measure the absorbance at 450 nm using a microplate reader, and we produced hemolytic activity curves by GraphPad prism 5.0.

### 4.11. Toxicity in 3D Tumor Spheroids

To further study the toxicity of LVTX-9 and LVTX-9-C_18_ in vitro, B16-F10 tumor spheroids, as 3D tumor models, were cultured according to protocols in the literature [39]. Briefly, B16-F10 cells were seeded into an ultra-low attachment, 96-well, round-bottom plate (Corning) and cultured at 37 °C for 3–4 days. Then, the B16-F10 tumor spheroids were treated with 50 or 25 μM of LVTX-9 and 20 or 10 μM of LVTX-9-C_18_ in the RPMI-1640 supplied with 10% serum for 3 days. Subsequently, images were taken on days 0 and 3, and the cell viability was measured using a CellTiter-Glo 3D Cell Viability Assay (G9683).

### 4.12. Statistical Analysis

Quantitative data are expressed as the mean ± SD. Statistical analyses were performed using GraphPad Prism software version 5.0. Data were analyzed by using the one-way ANOVA test or *t*-test (and nonparametric). The levels of significance were assigned as * *p* ≤ 0.05, ** *p* ≤ 0.01, and *** *p* ≤ 0.001.

## Figures and Tables

**Table 1:** Sequences, molecular masses, and retention times of peptides.

Peptides	Sequence	Mw (Da)	T_R_^a^ (min)
LVTX-9	NH2-ASIGALIQKAIALIKAKAA-CONH2	1851.28	15.21
LVTX-9-C_12_	CH3-(CH2)_10_-CONHASIGALIQKAIALIKAKAA-CONH2	2033.64	36.52
LVTX-9-C_14_	CH3-(CH2)_12_-CONHASIGALIQKAIALIKAKAA-CONH2	2061.65	36.15
LVTX-9-C_16_	CH3-(CH2)_14_-CONHASIGALIQKAIALIKAKAA-CONH2	2089.70	40.21
LVTX-9-C_18_	CH3-(CH2)_16_-CONHASIGALIQKAIALIKAKAA-CONH2	2117.76	41.89
LVTX-9-C_20_	CH3-(CH2)_18_-CONHASIGALIQKAIALIKAKAA-CONH2	2145.81	43.06

^a^ The retention time (T_R_) of each peptide was determined by the maximum height of the peaks from RP-HPLC, which indicates the hydrophobicity of the peptides.

**Table 2:** The cytotoxic activities (IC_50_) of LVTX-9 and LVTX-9-C_18_.

IC_50_ (μM)					
Peptides	L-929	A549	4T1	HepG2	Hela
LVTX-9	>100	51.7 ± 4.3	>100	>100	>100
LVTX-9-C_18_	3.6 ± 1.0	6.2 ± 1.4	7.6 ± 1.3	7.0 ± 1.0	4.7 ± 1.4

## Data Availability

Data sharing not applicable.

## Supplementary Materials

The following are available online at https://www.mdpi.com/article/10.3390/toxins13120867/s1, Figure S1: Cell viabilities of the peptides. Cytotoxic activities of of LVTX-9 and five lipopeptides on L-929 cells in serum-containing (A) or serum-free (B) medium as determined by CCK-8 assay, Table S1: Purification gradient of peptides.
