# Supplementary material for: Fatty Acid Modification of the Anticancer Peptide LVTX-9 to Enhance Its Cytotoxicity against Malignant Melanoma Cells
Source: Toxins (Basel). 2021 Dec 4;13(12):867. doi: 10.3390/toxins13120867 (PMC8708390; doi:10.3390/toxins13120867)
Supplement: Supplementary file 1 [file toxins-13-00867-s001.zip › toxins-1444629-supplementary.pdf]

Supplementary Materials: Fatty Acid Modification of the Anticancer Peptide LVTX-9 to Enhance Its Cytotoxicity against Malignant Melanoma Cells

Fengjiao Li, Saizhi Wu, Ninglin Chen, Jingyu Zhu, Xinxin Zhao, Peng Zhang, Youlin Zeng and Zhonghua Liu

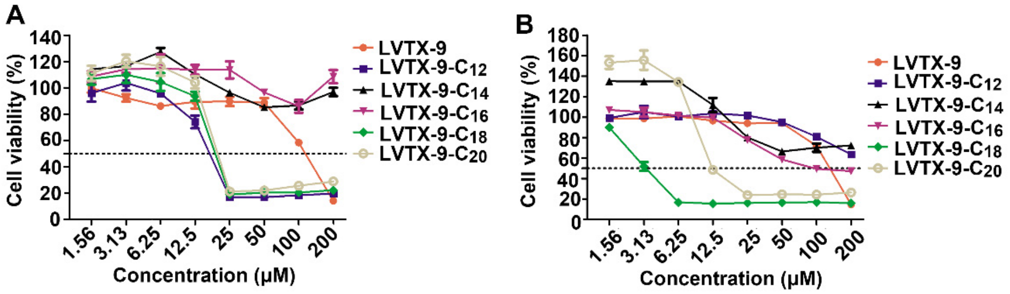

Figure S1. Cell viabilities of the peptides. Cytotoxic activities of of LVTX-9 and five lipopeptides on L-929 cells in serum-containing (**A**) or serum-free (**B**) medium as determined by CCK-8 assay.

Table S1. Purification gradient of peptides.

Time (min)	Flow Rate (mL/min)	A%	B%
0	1	80	20
4.9	1	80	20
5	1	70	30
40	1	0	100
40.1	1	0	100
43	1	0	100
43.1	1	80	20
46	1	80	20
46.1	0.1	80	20

Wavelength: 215 nm/280 nm, column temperature: 25 ± 5 °C, A: ddH_2O (0.1% TFA), B: acetonitrile (0.1% TFA).
